# Interstitial lung abnormalities in patients with stage I non-small cell lung cancer are associated with shorter overall survival: the Boston lung cancer study

**DOI:** 10.1186/s40644-021-00383-w

**Published:** 2021-01-19

**Authors:** Tomoyuki Hida, Akinori Hata, Junwei Lu, Vladimir I. Valtchinov, Takuya Hino, Mizuki Nishino, Hiroshi Honda, Noriyuki Tomiyama, David C. Christiani, Hiroto Hatabu

**Affiliations:** 1 Center for Pulmonary Functional Imaging, Department of Radiology, Brigham and Women’s Hospital and Harvard Medical School, 75 Francis St, Boston, MA 02115 USA; 2 Department of Clinical Radiology, Graduate School of Medical Sciences, Kyushu University, Fukuoka, Japan; 3 Department of Future Diagnostic Radiology, Osaka University Graduate School of Medicine, Osaka, Japan; 4 Department of Biostatistics, Harvard TH Chan School of Public Health, Boston, MA USA; 5 Department of Imaging, Dana Farber Cancer Institute, Boston, MA USA; 6 Department of Diagnostic and Interventional Radiology, Osaka University Graduate School of Medicine, Osaka, Japan; 7 Department of Environmental Health, Harvard TH Chan School of Public Health, Boston, MA USA; 8 Pulmonary and Critical Care Division, Department of Medicine, Massachusetts General Hospital/Harvard Medical School, Boston, MA USA

**Keywords:** Lung cancer, Interstitial lung abnormality, Computed tomography

## Abstract

**Background:**

Interstitial lung abnormalities (ILA) can be detected on computed tomography (CT) in lung cancer patients and have an association with mortality in advanced non-small cell lung cancer (NSCLC) patients. The aim of this study is to demonstrate the significance of ILA for mortality in patients with stage I NSCLC using Boston Lung Cancer Study cohort.

**Methods:**

Two hundred and thirty-one patients with stage I NSCLC from 2000 to 2011 were investigated in this retrospective study (median age, 69 years; 93 males, 138 females). ILA was scored on baseline CT scans prior to treatment using a 3-point scale (0 = no evidence of ILA, 1 = equivocal for ILA, 2 = ILA) by a sequential reading method. ILA score 2 was considered the presence of ILA. The difference of overall survival (OS) for patients with different ILA scores were tested via log-rank test and multivariate Cox proportional hazards models were used to estimate hazard ratios (HRs) including ILA score, age, sex, smoking status, and treatment as the confounding variables.

**Results:**

ILA was present in 22 out of 231 patients (9.5%) with stage I NSCLC. The presence of ILA was associated with shorter OS (patients with ILA score 2, median 3.85 years [95% confidence interval (CI): 3.36 – not reached (NR)]; patients with ILA score 0 or 1, median 10.16 years [95%CI: 8.65 - NR]; *P* <  0.0001). In a Cox proportional hazards model, the presence of ILA remained significant for increased risk for death (HR = 2.88, *P* = 0.005) after adjusting for age, sex, smoking and treatment.

**Conclusions:**

ILA was detected on CT in 9.5% of patients with stage I NSCLC. The presence of ILA was significantly associated with a shorter OS and could be an imaging marker of shorter survival in stage I NSCLC.

**Supplementary Information:**

The online version contains supplementary material available at 10.1186/s40644-021-00383-w.

## Background

Lung cancer is the most common cause of cancer related death in both men and women in the United States [1]. Clinicians including oncologists, and pulmonologists as well as epidemiologists and cancer patients have been interested in factors influencing the overall survival (OS). We have a very unique opportunity to study chest CT scans from The Boston Lung Cancer Study (BLCS; principal investigator [PI]: David C Christiani), which is a cancer epidemiology cohort of 11,164 lung cancer cases enrolled at Massachusetts General Hospital (MGH) and Dana-Farber Cancer Institute (DFCI) since 1992 with detailed demographic, smoking, occupational, and dietary information, in addition to pathology, imaging, treatments, oncogenic (somatic driver) mutation status, and bio-samples, which have been funded by National Institute of Health.

The importance of interstitial lung abnormalities (ILA) is increasingly recognized worldwide [2–11]. ILA is defined as radiologic patterns of increased lung density including non-dependent ground-glass or reticular abnormalities, diffuse centrilobular nodularity, non-emphysematous cysts, honeycombing, and traction bronchiectasis affecting more than 5% of any lung zone on chest computed tomography (CT) [8]. Although the pathological investigation of ILA has been limited, ILA may include an early and/or mild form of pulmonary fibrosis [8, 12]. Previous reports revealed that ILA is associated with increased respiratory symptoms, reductions of lung volume, exercise capacity, and gas exchange, and a greater risk of all-cause mortality [4, 5, 11, 13, 14]. ILA is relatively common, particularly in older individuals, but its presence is not routinely recorded on radiology reports, even at academic centres [2].

As low-dose lung cancer CT screening is performed commonly, early stage lung cancers are increasingly being detected. At the same time, ILA can be also detected on CT in those lung cancer patients. Our previous studies reported an association between the presence of ILA and mortality in advanced non-small cell lung cancer (NSCLC), which was confirmed in another cohort of stage IV NSCLC [15, 16]. However, we do not know whether presence of ILA on the baseline chest CT scans is associated with poorer OS in stage I NSCLC. Because stage I NSCLC patients survive longer compared to those with advanced NSCLC, it requires larger cohort with longer follow-up period to study this important question, which is now possible with BLCS.

We hypothesized that ILA have an association with mortality in patients with stage I NSCLC. The aim of this study is to demonstrate the significance of ILA for mortality in patients with stage I NSCLC using BLCS cohort.

## Methods

### Boston lung Cancer study (BLCS)

The Boston Lung Cancer Study (BLCS), PI: David C Christiani, is a cancer epidemiology cohort of 11,164 lung cancer cases enrolled at MGH and DFCI since 1992. The detail of this cohort was described previously [17]. This cohort has collected detailed demographic, smoking, occupational, and dietary information, in addition to pathology, imaging, treatments, oncogenic (somatic driver) mutation status, and bio-samples. The BLCS biorepository includes serum, white blood cells, DNA, and ~ 2000 tumor and surrounding tissues. MGH and DFCI are leaders in systematically genotyping patients for oncogenic mutations [18–25]. The construction of imaging cohort is described in the supplementary material.

### Patients

**Fig. 1:**
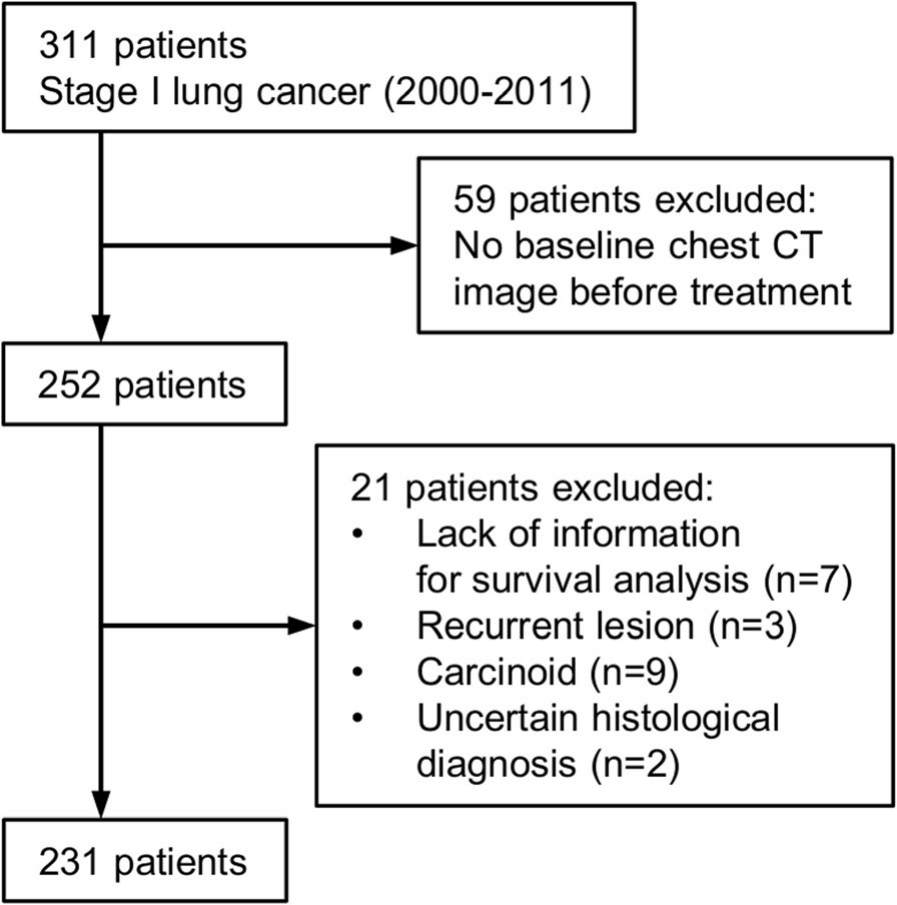
Flowchart of the selection and exclusion of the study population

Our IRB approved this retrospective cohort study. Three hundred and eleven patients, who were treated as stage I NSCLC (American Joint Committee on Cancer [AJCC] 7th edition) in Massachusetts General Hospital diagnosed between 2000 and 2011, were collected from the file of lung cancer cases of the institute (Fig. 1). For the patients before the publication of AJCC 7th, we converted all classification to the 7th. Two hundred and fifty-two out of 311 patients had a baseline chest CT image before treatment available for review. The cases with lack of information needed for survival analysis (no follow up, 5 cases; no smoking information, 2 cases) or who were treated for recurrent lesions (3 cases) were excluded from this study. Based on histology of surgical specimen, 9 cases of carcinoid and 2 cases with uncertain histological diagnosis were excluded. A total of 231 individuals were eligible for the analysis. Clinical record of the demographics including age, sex, smoking status, treatment, and survival, as well as CT studies were reviewed. The patients in the study provided written informed consent.

### CT scan of the chest

CT scan was performed using standard chest CT protocols at the time between 2000 and 2011 with or without administration of the intravenous contrast. Approximately two-thirds of the CT scans were obtained with contrast-enhancement reflecting the fact that the baseline study was performed as a part of staging studies.

For the purpose of ILA scoring, axial images reconstructed with a lung algorithm and slice thickness from 1.25 to 5 mm were reviewed on Picture Archiving Communication Systems (PACS) workstations (Vitrea, Canon Medical Systems Inc., Nasu, Japan) with a window level of − 700 Hounsfield unit (HU) and a window width of 1500 HU as previously described [9].

### Scoring of interstitial lung abnormality

**Fig. 2:**
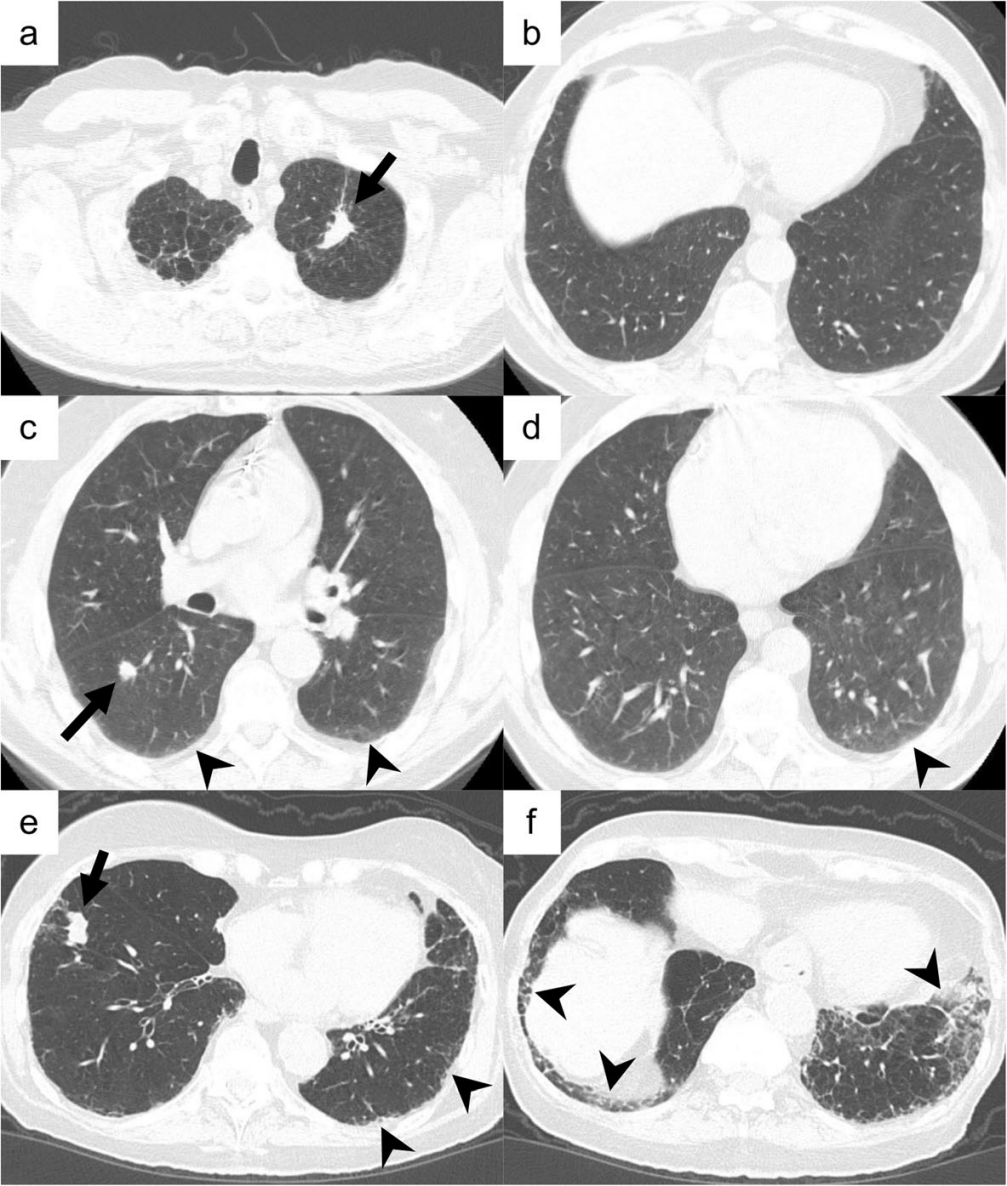
Examples of the ILA scoring. Axial CT images of three patients with lung cancer (arrows). **a**, **b** A 74-year-old male affected by squamous cell carcinoma of 2.5 cm in the left upper lobe (arrow). There was no interstitial lung abnormality (ILA; ILA score = 0). **c**, **d** A 64-year-old female affected by squamous cell carcinoma of 1.2 cm in the right lower lobe (arrow). The arrowheads demonstrate subpleural ground-glass opacity in less than 5% of the lung. The ILA score was 1 (equivocal for ILA) because it was unclear whether the finding was only dependent opacity or contained ILA. **e**, **f** A 84-year-old female affected by squamous cell carcinoma of 2.5 cm in the right lower lobe (arrow). The arrowheads point to subpleural ground-glass opacity and reticulation. These findings were seen in not only the dependent area but also in the ventral side. The ILA score was 2 (considered as ILA definitely)

The baseline chest CT images obtained at the time of diagnosis of lung cancer were reviewed retrospectively. Visual CT scoring of ILA was performed by using a sequential reading method previously described [4, 9]. ILA was scored on CT prior to each treatment using a 3-point scale [“0”, no evidence of ILA; “1”, equivocal for ILA; “2”, ILA]. ILA was defined as follows: nondependent ground glass abnormality affecting more than 5% of any lung zone, non-dependent reticular abnormality, diffuse centrilobular nodularity with ground-glass abnormality, honeycombing, traction bronchiectasis, non-emphysematous cysts, architectural distortion [4, 5, 7]. Equivocal for ILA (score of 1) was defined as focal or unilateral ground glass attenuation, focal or unilateral reticulation, and patchy ground-glass abnormality (less than 5% of the lung). Figure 2 shows examples of the ILA scoring.

In the sequential reading method, CT scans were reviewed by 3 board-certificate thoracic radiologists (T.H., H.H and M.N.). Reader 1 reviewed and scored all the CT studies of the cohort. Next, Reader 2 reviewed all the CT studies Reader 1 scored as 1 or 2, and randomly selected 20% of the studies Reader 1 scored as 0. Reader 2 reviewed these studies independently without knowing the scores of the Reader 1. If two readers gave concordant score, the studies received the concordant score as the final score. The final scores of the remaining 80% of the studies scored as 0 by Reader 1 were set as 0. The studies with discordant score by 2 readers were reviewed by Reader 3 independently and blindly. The majority opinion among three readers assigned as the final score of the study as described previously [4, 9]. For each reader, the CT scans were presented in a different random order.

### Statistical analysis

All statistical analyses were performed using R (Version 4.0.2, R Project for Statistical Computing). We investigate the relationship between the ILA score and the survival (OS) along with the clinical record including the age, sex, smoking status, and treatment. The treatment information includes whether the patients have taken surgery, chemotherapy or radiation therapy. All the clinical variables above were used as confounding variable in the statistical analysis.

OS was defined as the defined from the time of diagnosis to the death time from any cause. Patients who did not die by the time of analyses were censored at the last known date of follow-up. We used the multivariate Cox proportional hazards models to estimate hazard ratios (HRs) including both ILA score as well as age, sex, smoking status, and the treatment information as the confounding variables because they are common factors for the mortality in the lung cancer population. The difference of OS for patients with different ILA scores were tested via log-rank test. The difference in demographics and clinical records were assessed via the Fisher exact test for categorical data and the Wilcoxon test for continuous data. We evaluate all *P*-values at the significance level of 0.05.

## Results

The demographics of the patients and disease characteristics of the study population, and the ILA scores on the baseline chest CT studied were summarized in Table 1. Median follow-up time was 966 days (range, 12–5677 days). ILA (ILA score = 2) was present in 22 out of 231 cases with stage I NSCLC (9.5%) on CT prior to each treatment. Sixty-three cases showed equivocal ILA (ILA score = 1) and the other 146 cases had no evidence of ILA (ILA score = 0). Twenty-two patients with ILA score 2 were significantly older than patients with ILA score 0 or 1 (median age: 67 vs. 72, *P = 0.03*). Those with ILA score 2 had significantly more smoking history (never smoker vs. current/ex-smoker) and pack years of smoking than those with ILA score 0 or 1 (median pack years of smoking: 31.6 vs. 54.2, *P* <  0.001). Among the 231 patients, 52 (22.5%) patients died of any cause (39 patients with ILA score 0 or 1, 13 patients with ILA score 2).

**Table 1:** Demographics and ILA scores of the population

		ILA score			
		0 or 1 (*n* = 209)	2 (*n* = 22)	Total (*n* = 231)	*P* value
Age (years)	Median [range]	67 [40–91]	72 [58–84]	69 [40–91]	0.03^b^
Sex	Male	81 (38.8)	12 (54.5)	93 (40.3)	0.17
	Female	128 (61.2)	10 (45.5)	138 (59.7)	
Smoking	Never	35 (16.7)	0 (0.0)	35 (15.2)	0.03^c^
	Former	101 (48.3)	13 (59.1)	114 (49.4)	
	Current	73 (34.9)	9 (40.9)	82 (35.5)	
Pack years of smoking	Median [range]	31.6 [0.0–138.1]	54.2 [5.9–144.6]	34.5 [0.0–144.6]	< 0.001^b^
Histology	Adeno	143 (68.4)	5 (22.7)	148 (64.1)	< 0.001^c^
	Squamous	35 (16.7)	14 (63.6)	49 (21.2)	
	Other^a^	31 (14.8)	3 (13.6)	34 (14.7)	
Treatment	Surgery	194 (92.8)	18 (81.8)	212 (91.8)	
	Chemotherapy	27 (12.9)	3 (7.3)	30 (13.0)	
	Radiation	5 (2.4)	4 (18.2)	9 (3.9)	

Continuous values were presented as median [range]

Values for the number of patients are given as absolute number and percentage in parenthesis

^a^Includes large cell carcinoma, not otherwise specified, mixed tumors, and more than 1 primary tumors

^b^Significant differences between the two groups were calculated using Wilcoxon test

^c^Significant differences were calculated using Fisher’s exact test

*ILA* interstitial lung abnormalities

**Fig. 3:**
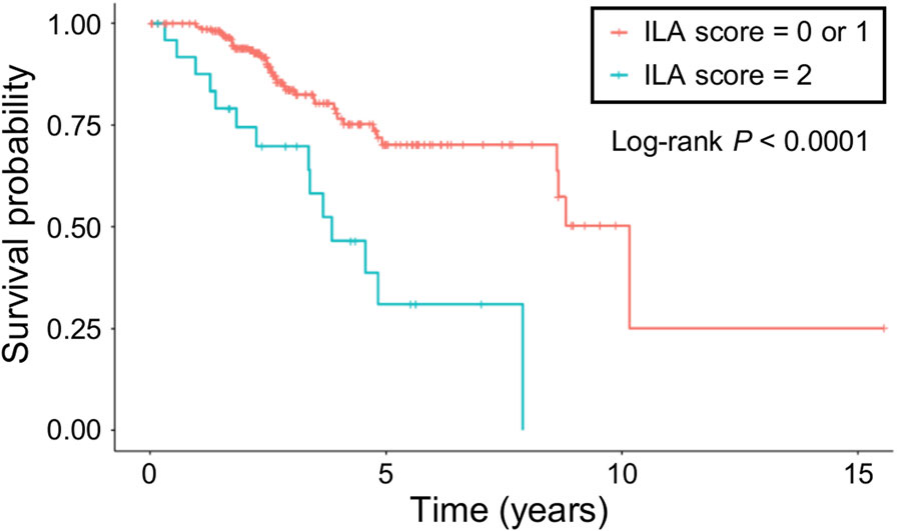
Overall survival of patients with and without ILA. The graph shows overall survival (OS) of patients without interstitial lung abnormalities (ILAs; ILA score = 0 or 1) and with ILA (ILA score = 2) using Kaplan-Meier estimate. Patients with ILA had significantly shorter OS than those without ILA

The presence of ILA was associated with shorter overall survival (OS) and the median OS of the 22 patients with ILA was median 3.85 years [95% confidence interval (CI): 3.36 – not reached (NR)] compared to median 10.16 years [95%CI: 8.65 - NR] in patients with ILA score 0 or 1 (*P* <  0.0001, Fig. 3). In a Cox proportional hazards model, the presence of ILA remained significant for increased risk for death (HR = 2.88, *P =* 0.005, Table 2) after adjusting for age, sex, smoking (never vs. current/ex-smoker) and treatment (surgery, chemotherapy and radiation therapy). Increasing age and use of chemotherapy was associated with increased risk of death, while surgical treatment was associated with decreased risk of death (age, HR = 1.03, *P* = 0.028; chemotherapy, HR = 2.57, *P* = 0.010; surgical treatment, HR = 0.21, *P* <  0.001). The concordance index for the Cox proportional hazards model was 0.737.

**Table 2:** The results for risk factors associated with OS in the Cox proportional hazards model

Characteristic	HR	95%CI	*P* value
ILA (score 2)^a^	2.88	1.37–6.04	0.005
Age	1.03	1.00–1.07	0.028
Sex (male)	0.62	0.33–1.16	0.135
Smoking status (never smoker)^b^	2.18	0.66–7.17	0.200
Surgery	0.21	0.09–0.50	< 0.001
Chemotherapy	2.57	1.26–5.27	0.010
Radiation therapy	0.96	0.31–2.98	0.954

The concordance index for the Cox proportional hazards model was 0.737

^a^Comparison is ILA score 2 with ILA score 0 or 1

^b^Comparison is never smoker with current/ex-smoker

*OS* overall survival, *ILA* interstitial lung abnormalities, *HR* hazard ratio, *CI* confidence interval

## Discussion

To our knowledge, this is the first report that revealed the association between ILAs and OS in stage I NSCLC patients. ILA was present in 9.5% of stage I NSCLC patients on CT prior to treatment. Patients with ILA at diagnosis had significantly shorter overall survival as well as patients with stage IV NSCLC [15, 16].

Previous studies reported that pulmonary fibrosis and/or ILAs were important factors for worse prognosis in patients with early stage lung cancer. Iwasawa et al. investigated 217 patients with stage I and II cancers and showed that existence of ILAs predicted poorer disease-free survival (DFS; HR, 3.3; *P* <  0.001) [26]. Im et al. investigated 488 patients who underwent curative resections for stage I and II cancers and showed that ILA was independently associated with postoperative pulmonary complications and four patients died at 180 days after surgery due to respiratory failure, aspiration pneumonia, and empyema [27]. Saito et al. also investigated 350 patients with stage IA lung cancer treated with pulmonary resection and revealed that the 5-year survival rates were 54.2% in patients with idiopathic pulmonary fibrosis (IPF) and 88.3% in those without IPF (*P* <  0.0001) [28]. They concluded that IPF was an independent factor for worse survival. Our results are consistent with these previous results.

The specific causes of death remain unclear because it was unavailable in our cohort. However, the shorter OS in patient with ILA are likely partially explained by respiratory causes and cancer recurrence. Putman et al. reported that participants with ILA were more likely to die of a respiratory cause than those without ILA in AGES-Reykjavik cohort study (odds ratio, 2.4; *P* < 0.001) [8]. Saito et al. showed that the rates of cancer-related death were 17.9% for stage IA lung cancer patients with IPF and 3.7% for those without IPF (*P* < 0.001) and the rates of deaths due to respiratory failure were 14.3% for those with IPF and 1.2% for those without IPF (*P* < 0.0001) [28]. It should be noted, however, that they also performed propensity score-matching analysis and showed that the difference in the cancer-related death was not significant in the matched analysis. In the study of Iwasawa et al., eleven (44%) of 25 patients with IPF died because of cancer recurrence (*n* = 5) and IPF (*n* = 6) [26].

Unlike advanced lung cancer, few patients with early stage cancer receive chemotherapy. In the previous studies which investigated the association between ILA and advanced cancer, drug induced pneumonitis was considered as an important factor which resulted in short OS [15, 16]. Fujimoto et al. reported that 6% of patients (44/770) showed pneumonitis during the therapy and preexisting interstitial lung disease was independently associated with higher incidence of pneumonitis [29]. Further investigations are necessary to reveal why patients with ILA have shorter survival among lung cancer patients.

The prevalence of ILA was 9.5% in stage I NSCLC patients in this study, which is similar to the results of previous general cohort studies. It has been reported that the prevalence of ILA was 7% in Framingham Heart Study, 7% in AGES-Reykjavik, 8% in COPDGene, 9% in ECLIPSE, and 9.7% in NLST [6, 8]. In lung cancer patients, the prevalence of ILA varies from 3.8 to 21.7% among different cohort [15, 16, 26, 29]. The prevalence may be influenced by multiple factors including interpretations by radiologists. Iwasawa et al. used both CT evaluation by radiologist and computer-aided detection (CAD) system to assess pulmonary fibrosis and reported that the CAD result was correlated with the fibrosis extent determined by radiologists and significantly predicted worse DFS of lung cancer [26]. In the near future, quantitative methods may be useful options to evaluate ILA with high reproducibility.

The prevalence of squamous cell carcinoma was substantially higher in the ILA group (63.6% in ILA group vs. 16.7% in non-ILA group). Iwasawa et al. also reported that ILA group showed higher prevalence of squamous cell carcinoma than non-ILA group among early stage lung cancer patients (40.4% in ILA group vs. 7.0% in non-ILA group) [26]. On the other hands, among stage IV NSCLC patients, there was no significant difference in histology prevalence in previous reports [15, 16]. As both squamous cell carcinoma and ILA are associated with smoking [8], the high prevalence in ILA group among early stage patients is reasonable. However, the reason for the discrepancy between early and advanced stage cancer is unclear. The difference in patient characteristics and selection bias may result in the discrepancy.

This study has some limitations. First, this study was retrospective design with patients treated at a limited number of institutions. The results of the study need to be validated in a larger cohort. In addition, the population of the patients with ILA was relatively small, although the results are highly statistically significant. Second, this study includes both CT images with or without intravenous contrast agent. The difference might have influence on scoring of ILA, although it is probably negligible considering the very wide window width setting of 1500 HU. Also, the application of contrast would not be expected to introduce bias unless contrast was applied differentially to those with and without ILA, which did not occur here. Third, histological confirmation of ILA was not obtained in this study. Fourth, pulmonary function test data and patient performance like six-minute walk test were not analyzed in this study. Fifth, high resolution CT protocol in prone position was not used in this study. Sixth, subcategories (non-subpleural, subpleural non-fibrotic, and subpleural fibrotic) and quantification of ILA were not evaluated in this study. In addition, the other factors such as emphysema, known interstitial lung disease, and history of other malignancies were not assessed. There should be the combination of ILA and emphysema because both ILA and emphysema are common in smoking patients. Further investigations for these aspects may provide useful information for the management of lung cancers. Last, we used AJCC 7th edition for lung cancer staging criteria, because staging by the latest version (AJCC 8th edition) was not available in our cohort. Staging by the latest version may result in some effect on survival rate from that in our results. 

## Conclusions

ILA was detected on CT in 9.5% of patients with stage I NSCLC. The presence of ILA was significantly associated with a shorter OS and could be an imaging marker of shorter survival in stage I NSCLC.

## Supplementary Information

**Additional file 1.**

## Data Availability

Not applicable.

## Abbreviations

AJCC: American Joint Committee on Cancer
BCLS: The Boston Lung Cancer Study
CI: Confidence Interval
DICOM: Digital Imaging and Communications in Medicine
HR: Hazard Ratio
ILA: Interstitial Lung Abnormalities
IPF: Idiopathic Pulmonary Fibrosis
NSCLC: Non-Small Cell Lung Cancer
NR: Not Reached
OS: Overall Survival
