# Supplementary material for: Interstitial lung abnormalities in patients with stage I non-small cell lung cancer are associated with shorter overall survival: the Boston lung cancer study
Source: Cancer Imaging. 2021 Jan 19;21:14. doi: 10.1186/s40644-021-00383-w (PMC7816399; doi:10.1186/s40644-021-00383-w)
Supplement: Supplementary file 1 — Additional file 1. [file 40644_2021_383_MOESM1_ESM.docx]

**Construction of Imaging Cohort for the Boston Lung Cancer Study (BLCS)**

For the last 18 months, an additional sub-cohort of the BLCS was being built, PI. David Christiani, co-investigator (co-I): Hiroto Hatabu. It contains full-fidelity diagnostic images of CT of the Chest, or Positron Emission (PE) of the Chest imaging for a large majority of the subjects in BLCS. Below we spell out the construction steps for future references.

Step #1: A trained medical imaging analyst reviewed all 11, 614 cases present in the BLCS. An institutional review board (IRB) approval was obtained from Partners (protocol # 1999P004935/PHS) was obtained to allow access to protected health information data on all patients consented for the BLCS study. Patient Lookup was performed in the Enterprise-wide Electronic Medical System from Epic (Verona, Wisconsin, United States). In batches of 500, all medical record numbers (MRNs) of the subjects containing Chest CT or Chest PE imaging were recorded and stored.

Step #2: Access to the enterprise research system at Partners Healthcare, Inc., Research Patient Data Repository (RPDR) was obtained by same IRB. Requests for retrieval of the associated images for the MRNs recorded were submitted to RPDR.

Step #3: We have further used the mi2b2 system to retrieve and persist the full-fidelity Digital Imaging and Communications in Medicine (DICOM) images do disk, using DICOM-Dir structure.

Step #4: In addition, the final repository of chest CT and chest PE images was de-identified using a direct, sequential MRN-to-new-identifier mapping. All Private Health Information fields from the DICOM header were deleted except for a newly generated set of keys. The master mapping file is updated and stored away by the PI and co-I on physically separate computer systems.
